# Application of donor predicted heart mass in heart transplant recipients with left ventricular assist device

**DOI:** 10.1016/j.jhlto.2024.100150

**Published:** 2024-08-22

**Authors:** Ross M. Reul, Qiudong Chen, Joshua L. Chan

**Affiliations:** a Division of Cardiothoracic Surgery, Department of Surgery, Emory University School of Medicine, Atlanta, Georgia; b Department of Cardiac Surgery, Smidt Heart Institute, Cedars Sinai Medical Center, Los Angeles, California

**Keywords:** heart transplant, mechanical circulatory support, left ventricular assist device, bridge-to-transplantation, predicted heart mass

## Abstract

**Background:**

An association between predicted heart mass (PHM) and post heart transplantation outcomes has been well established; however, there is limited data on the effect of donor PHM on recipients bridged with a durable left ventricular assist device (LVAD). This retrospective observational study seeks to challenge the theoretical benefit of oversizing donor hearts for recipients bridged with LVADs.

**Methods:**

Analysis of the United Network for Organ Sharing database revealed 10,806 adult patients with 1 of 3 durable LVADs (HeartMate 2, HeartMate 3 [HM3], HeartWare Ventricular Assist Device) between January 1, 2015 and December 31, 2021. Baseline characteristics were compared between 7 equally sized groups based on donor-to-recipient PHM. Univariable and multivariable Cox regression analyses were constructed to evaluate the effect of PHM size-matching on the primary outcome of 1-year post-transplant survival. Further analyses were performed specifically on HM3 patients and with PHM as a continuous variable.

**Results:**

Multivariable analysis revealed that severely undersized donor hearts (PHM ratio <0.86) resulted in worse outcomes with respect to 1-year mortality (hazard ratio 1.30; confidence interval 1.03-1.64, *p* = 0.03). There was no significant benefit to oversizing donor hearts. Similar results were found in patients bridged to transplant with HM3.

**Conclusions:**

Similar to prior studies on heart transplant recipients, recipients bridged with durable LVAD had worse outcomes when using severely undersized donor hearts. Oversized donor hearts did not significantly improve 1-year mortality, compared to size-matched references. These results were consistent in a subgroup analysis of patients bridged only with HM3 LVADs.

## Background

Previous studies have established that predicted heart mass (PHM) provides the most optimal metric for size match in heart transplantation (HTX) compared to using height, weight, body surface area, or body mass index.^1^ Consequently, society guidelines now include a new recommendation to use PHM calculations in guiding donor selection (Class IIa, Level C).^2^ Despite an abundance of data showing the mortality benefit of optimal size-matching in HTX, the optimal size-matching strategy remains unclear for the population of patients who are bridged to transplantation with durable left ventricular assist devices (LVAD). It is thought that implanting oversized donor hearts may be beneficial in patients bridged to transplantation with durable LVADs. ^3^ This is in part based on body mass index size-matching data in the LVAD population, and speculatively, the relative consideration that a higher cardiac output provided by the oversized donor heart may be necessary to overcome the inherent vasoplegia and cytokine release associated with redo sternotomy and explantation of previously implanted durable mechanical circulatory support (MCS) device at the time of transplantation.^4^ However, no study has definitively demonstrated this relationship using donor-to-recipient PHM ratio. Additionally, the practice of choosing oversized donor hearts for these patients may further limit the optimal utilization of donor hearts in light of continued graft availability. Therefore, we sought to analyze the effect of size matching (as evaluated by the donor-to-recipient PHM ratio) on post-transplant outcomes in patients bridged to transplantation with a durable LVAD.

## Materials and methods

### Study population

This retrospective analysis was performed using the United Network for Organ Sharing (UNOS) Standard Transplant Analysis and Research files as of April 2023, which included information on organ donation, transplants, and new listings occurring through March 2023. We retrospectively identified all adult HTX recipients transplanted between January 1, 2015 and December 31, 2021 (*n* = 39,952).

Recipients with a history of previous heart transplant (*n* = 1,004), multiorgan transplant (*n* = 2,556), body mass index >40 (*n* = 242), and missing age, weight, or height (thus precluding calculation of the PHM, *n* = 14) were excluded from the analysis. Recipients were included in the analysis if they were bridged to heart transplant with a HeartMate 2, HeartMate 3 (HM3), or HeartWare Ventricular Assist Device. The final study population included 10,806 adult heart transplant recipients bridged to transplant with 1 of the 3 included durable LVADs. HTX recipients were divided into 7 equal groups based on the donor-to-recipient PHM ratio (Table 1), similar to the seminal PHM analysis by Kransdorf et al.^1^

**Table 1:** Cutoff Values for Donor-to-Recipient Predicated Heart Mass Ratio After the Cohort is Divided into 7 Equal Groups

Group	Number of patients	Median donor-to-recipient PHM ratio	Mean donor-to-recipient PHM ratio	Minimum donor-to-recipient PHM ratio	Maximum donor-to-recipient PHM ratio
Severely undersized	1,543	0.81	0.80	0.55	0.86
Moderately undersized	1,544	0.89	0.89	0.86	0.92
Mildly undersized	1,544	0.94	0.94	0.92	0.96
Matched	1,544	0.99	0.99	0.96	1.01
Mildly oversized	1,544	1.04	1.04	1.01	1.08
Moderately oversized	1,544	1.12	1.12	1.08	1.17
Severely oversized	1,543	1.26	1.29	1.17	1.95

Abbreviation: PHM, predicted heart mass.

The overall cohort was divided into 7 equal groups based on the donor-to-recipient predicted heart mass ratio.

Recipient/donor characteristics and patient outcomes were defined according to the standard UNOS definitions. Those with UNOS status 1A before the 2018 allocation policy change and status 1, 2, or 3 afterward were considered to have urgent status at transplant. Donor-to-recipient PHM ratio was calculated with a previously developed formula using recipient age, sex, height, and weight, and was used as a surrogate for donor-recipient size match.^5^, ^6^ Recipient functional status was classified using the Karnofsky Performance Scale Index. Due to the deidentified nature of the database, this study is exempt from Institutional Review Board approval and in compliance with the International Society for Heart and Lung Transplantation ethics statement, and informed consent was not necessary.

### Primary and secondary outcomes

The primary outcome was 1-year survival after HTX. Because survival alone may inadequately reflect post-transplant outcomes, we also evaluated the association between donor-recipient PHM ratio and a secondary composite end-point of textbook outcome (available only in 8,999 patients with complete 1-year follow-up data). This was defined as post-transplant hospital length of stay <30 days; ejection fraction greater than 50% during 1-year follow-up; functional status 80% to 100% at 1 year; freedom from acute rejection, dialysis, and stroke during the index hospitalization; and freedom from graft failure, dialysis, rejection, retransplantation, or mortality during the first year after transplantation. This composite end-point was chosen because previous work has demonstrated that the use of textbook outcome as an adjunctive metric provides a more holistic view of patient outcomes after HTX.^7^

### Statistical analysis

Recipients were divided into 7 equal groups based on the donor-to-recipient PHM ratio. Baseline characteristics were described either as means ± standard deviation or as medians and interquartile range for continuous variables depending on the overall distribution. Categorical variables were described as frequencies and percentages. Differences between groups were analyzed using Student’s *t*-test or Wilcoxon signed-rank test for continuous variables depending on the overall distribution. Pearson’s chi-square or Fisher exact was performed for categorical variables where appropriate. Post-transplant survival was analyzed using the Kaplan-Meier method and compared using the log-rank test. The multiple-comparison adjustment was performed, and paired comparisons were used, using the appropriately size-matched group (donor/recipient PHM ratio between 0.964 and 1.103) as the reference.

A multivariable Cox regression was constructed to evaluate the independent effect of the donor-to-recipient PHM ratio on 1-year post-transplant mortality. Covariates included in the models were selected a priori based on clinical relevance. These included recipient characteristics (race, age, etiology of heart failure, mean pulmonary artery pressure [pulmonary vascular resistance is not a variable in the UNOS database], pretransplant dialysis, diabetes, cerebrovascular disease, previous cardiac surgery, listing status, pretransplant MCS, mechanical ventilation, functional status, hospitalization status, era of transplant, baseline serum creatinine, and total bilirubin) and donor characteristics (age, sex-mismatch status, left ventricular function, and total ischemic time). The clustering of patients within each transplant center was accounted for using a robust variance estimator. The proportional hazard assumption was also checked with martingale residuals. The donor-to-recipient PHM ratio was first incorporated as a categorical variable with 7 equal groups, and patients with appropriate donor-to-recipient size match (0.964 < D/R PHM ratio < 1.013) were chosen as the reference group for comparison. Next, we examined the effect of donor-to-recipient PHM ratio as a continuous variable by modeling it using restricted cubic splines with 5 knots placed at the 5th, 27.5th, 50th, 72.5th, and 95th percentiles of the data, and a donor-to-recipient PHM ratio of 1 (suggesting perfect size match) was set as the reference value for comparison. Similar analyses were performed using the subgroup of patients bridged to transplantation with HM3 only. In this subgroup of patients, recipients were also divided into 7 equal groups based on the donor-to-recipient PHM ratio (Table S1).

We also constructed a multivariable logistic regression model to assess the association between the donor-to-recipient PHM ratio and the composite end-point of the “textbook outcome” (including hospital length of stay, ejection fraction, functional status, and perioperative and 1-year morbidity) using 8,999 patients from the initial cohort with available 1-year follow-up data.^7^ The covariates included in this model were similar to those included in the Cox model, as mentioned in the preceding text. A multivariable generalized estimating equation model with a logit link function and binomial distribution was fitted, adjusting for clustering at a center level. The donor-to-recipient PHM ratio was incorporated as a categorical variable with 7 equal groups, and patients with appropriate donor-to-recipient size match were chosen as the reference group for comparison. Similar analyses were also performed using the subgroup of patients bridged to transplantation with HM3 only.

All tests were 2-tailed with an alpha level of 0.05. All statistical analyses were performed using SAS 9.4 (SAS Institute, Cary, NC).

## Results

### Study population

A total of 10,608 HTX recipients were bridged to transplant with a durable LVAD, including 1,267 bridged with HM3. These patients were evenly distributed into 7 cohorts based on the donor-to-recipient PHM ratio (Table 1). Baseline donor and recipient characteristics stratified by PHM ratio cohort are listed in Table 2. Baseline characteristics were similar between PHM cohorts for the majority of queried factors. There was a higher proportion of undersizing in recipients with male sex, White race, diabetes, and elevated creatinine. Both severely undersized and severely oversized patients spent less average time on the waitlist. Recipients of male donor hearts were more likely to be oversized. Donors at both extremes were more likely to be older and had higher numerical rates of diabetes and hypertension.

**Table 2:** Baseline Recipient and Donor Characteristics Stratified by Degree of Size Mismatch Based on Donor-to-Recipient Predicted Heart Mass Ratio

Recipient and donor characteristics	Severely undersized (*n* = 1,543)	Moderately undersized (*n* = 1,544)	Mildly undersized (*n* = 1,544)	Matched (*n* = 1,544)	Mildly oversized (*n* = 1,544)	Moderately oversized (*n* = 1,544)	Severely oversized (*n* = 1,543)	*p*-value
*Recipient variables*								
Age (years)	54 (45-62)	55 (45-62)	57 (49-64)	57 (48-63)	57 (48-63)	57 (48-64)	57 (48-63)	<0.001
Male sex	94.3 (1,455)	89.5 (1,382)	88.5 (1,367)	84.6 (1,306)	80.1 (1,236)	73.9 (1,141)	50.4 (778)	<0.001
White race	63.4 (978)	68.5 (1,058)	65.2 (1,007)	63.2 (975)	65.0 (1,004)	62.6 (967)	62.0 (957)	0.002
Diabetes	34.9 (539)	31.5 (487)	33.2 (513)	31.8 (491)	30.6 (472)	28.0 (432)	27.3 (421)	<0.001
Cerebrovascular disease	5.9 (91)	6.2 (96)	6.7 (104)	5.6 (87)	7.5 (116)	6.9 (106)	6.6 (102)	0.41
Etiology of heart failure								0.05
Dilated cardiomyopathy	48.8 (753)	49.4 (762)	45.7 (706)	47.3 (731)	49.2 (759)	47.7 (736)	52.0 (802)	
Ischemic cardiomyopathy	38.3 (591)	36.9 (570)	41.3 (638)	40.0 (617)	37.9 (585)	38.2 (590)	33.5 (517)	
Restrictive/hypertrophic cardiomyopathy	0.9 (14)	1.4 (22)	0.8 (12)	0.7 (11)	1.0 (16)	1.2 (18)	0.9 (14)	
Congenital heart disease	0.4 (6)	0.7 (10)	0.7 (10)	0.8 (12)	1.0 (16)	1.0 (15)	0.7 (11)	
Others	11.6 (179)	11.7 (180)	11.5 (178)	11.2 (173)	10.9 (168)	12.0 (185)	12.9 (199)	
Mean pulmonary artery pressure >25 mm Hg	48.7 (752)	45.9 (709)	45.1 (697)	47.8 (738)	44.6 (688)	46.1 (711)	46.5 (718)	0.24
Creatinine	1.20 (1.00-1.50)	1.20 (1.00-1.48)	1.20 (1.00-1.46)	1.20 (1.00-1.45)	1.18 (0.97-1.40)	1.13 (0.93-1.40)	1.10 (0.89-1.38)	<0.001
Total bilirubin	0.7 (0.5-1.0)	0.7 (0.5-1.0)	0.7 (0.5-1.0)	0.6 (0.5-1.0)	0.6 (0.4-0.9)	0.7 (0.4-1.0)	0.6 (0.4-0.9)	0.001
Pretransplant dialysis	2.3 (35)	1.9 (30)	1.4 (22)	1.2 (19)	1.6 (24)	2.0 (31)	1.5 (23)	0.26
Pretransplant ECMO	0.3 (5)	0.1 (2)	0.2 (3)	0.3 (5)	0.4 (6)	0.3 (4)	0.3 (4)	0.85
Pretransplant IABP	0.7 (10)	0.6 (9)	0.3 (4)	0.2 (3)	1.1 (17)	1.0 (16)	0.5 (8)	0.006
Pretransplant mechanical ventilation	0.5 (8)	0.4 (6)	0.5 (7)	0.2 (3)	0.8 (12)	0.3 (5)	0.3 (4)	0.21
Urgent status at transplant^a^	68.3 (1,054)	68.9 (1,064)	66.5 (1,026)	70.0 (1,081)	69.4 (1,072)	71.7 (1,107)	73.0 (1,127)	0.002
Days on waiting list	187 (57-440)	217 (78-484)	216 (89-496)	226 (75-493)	204 (66-469)	198 (72-439)	162 (59-367)	<0.001
Pretransplant hospitalization status								<0.001
Hospitalized, ICU	9.7 (149)	7.8 (120)	7.1 (109)	7.4 (114)	8.6 (133)	7.7 (119)	10.3 (159)	
Hospitalized, non-ICU	13.3 (205)	15.4 (238)	11.3 (175)	14.9 (230)	13.5 (209)	15.8 (244)	15.3 (236)	
Not hospitalized	77.1 (1,189)	76.8 (1,186)	81.6 (1,260)	77.7 (1,200)	77.9 (1,202)	76.5 (1,181)	74.4 (1,148)	
Transplant era								<0.001
2005-2010	13.7 (212)	9.7 (149)	11.4 (176)	11.6 (179)	11.3 (174)	14.2 (219)	14.1 (218)	
2011-2016	44.8 (691)	46.8 (722)	48.2 (744)	46.6 (719)	49.4 (762)	48.2 (744)	48.4 (747)	
2017-2021	41.5 (640)	43.6 (673)	40.4 (624)	41.8 (646)	39.4 (608)	37.6 (581)	37.5 (578)	
Pretransplant function status								0.27
Mild dysfunction	20.7 (320)	19.1 (295)	18.8 (290)	20.6 (318)	21.2 (328)	21.3 (329)	18.8 (290)	
Moderate dysfunction	40.8 (629)	42.2 (652)	42.9 (663)	41.3 (637)	41.5 (640)	40.1 (619)	39.5 (610)	
Severe dysfunction	34.3 (528)	34.2 (528)	32.7 (505)	33.4 (516)	32.5 (502)	34.1 (527)	37.5 (579)	
Unknown	4.3 (66)	4.5 (69)	5.6 (86)	4.7 (73)	4.8 (74)	4.5 (69)	4.2 (64)	
*Donor variables*								
Age (years)	33 (24-42)	26 (23-29)	26 (23-29)	27 (24-30)	30 (23-39)	30 (22-38)	30 (22-38)	<0.001
Male sex	50.0 (771)	74.6 (1,152)	79.8 (1,232)	81.4 (1,257)	81.3 (1,255)	83.0 (1,282)	84.4 (1,302)	<0.001
Sex matching								<0.001
Female donor to female recipient	5.6 (87)	10.2 (157)	10.7 (165)	13.0 (200)	14.4 (222)	15.0 (231)	14.1 (218)	
Female donor to male recipient	44.4 (685)	15.2 (235)	9.5 (147)	5.6 (87)	4.3 (67)	2.0 (31)	1.5 (23)	
Male donor to female recipient	0.1 (1)	0.3 (5)	0.8 (12)	2.5 (38)	5.6 (86)	11.1 (172)	35.5 (547)	
Male donor to male recipient	49.9 (770)	74.3 (1,147)	79.0 (1,220)	79.0 (1,219)	75.7 (1,169)	71.9 (1,110)	48.9 (755)	
Diabetes	4.7 (73)	4.3 (66)	4.6 (71)	3.6 (56)	3.4 (53)	3.6 (55)	5.0 (77)	0.16
Hypertension	16.9 (261)	15.0 (231)	13.5 (209)	13.4 (207)	14.8 (229)	15.7 (242)	16.7 (258)	0.03
LVEF<50%	1.8 (27)	1.3 (20)	1.4 (21)	1.8 (28)	1.6 (24)	1.9 (29)	1.8 (27)	0.80
Ischemic time	3.28 (2.57-3.90)	3.25 (2.48-3.90)	3.18 (2.46-3.83)	3.25 (2.50-3.88)	3.23 (2.40-3.87)	3.25 (2.45-3.87)	3.15 (2.38-3.80)	0.03

Abbreviations: ECMO, extracorporeal membrane oxygenation; IABP, intra-aortic balloon pump; ICU, intensive care unit; LVEF, left ventricular ejection fraction.

a Urgent status at transplant = status 1A under the old allocation system or status 1, 2, or 3 under the new allocation system.

The unadjusted in-hospital post-transplant outcomes stratified by the donor-to-recipient PHM ratio are represented in Table 3. There were no significant differences in rates of dialysis, pacemaker implantation, stroke, or acute rejection. There was a significantly increased length of stay in undersized patients.

**Table 3:** In-Hospital Outcomes After Heart Transplantation Stratified by Degree of Size Mismatch Based on Donor-to-Recipient Predicted Heart Mass Ratio

Recipient and donor characteristics	Severely undersized (*n* = 1,543)	Moderately undersized (*n* = 1,544)	Mildly undersized (*n* = 1,544)	Matched (*n* = 1,544)	Mildly oversized (*n* = 1,544)	Moderately oversized (*n* = 1,544)	Severely oversized (*n* = 1,543)	*p*-value
Dialysis		14.6 (225)	12.1 (191)	12.4 (191)	12.3 (190)	11.3 (175)	12.6 (195)	0.12
Permanent pacemaker implant	2.7 (42)	2.7 (42)	3.0 (47)	3.8 (58)	3.2 (50)	2.6 (41)	3.1 (47)	0.58
Stroke	4.2 (65)	3.6 (55)	3.6 (56)	3.8 (59)	3.8 (58)	4.2 (65)	4.3 (67)	0.88
Acute rejection	20.8 (1,222)	21.8 (336)	19.4 (299)	19.7 (304)	20.3 (313)	18.4 (284)	18.7 (289)	0.23
Hospital length of stay (days)	17 (12-27)	16 (12-24)	16 (11-23)	16 (12-25)	16 (11-23)	15 (11-24)	15 (11-24)	<0.001

### Association between size matching and 1-year post-transplant mortality

**Figure 1:**
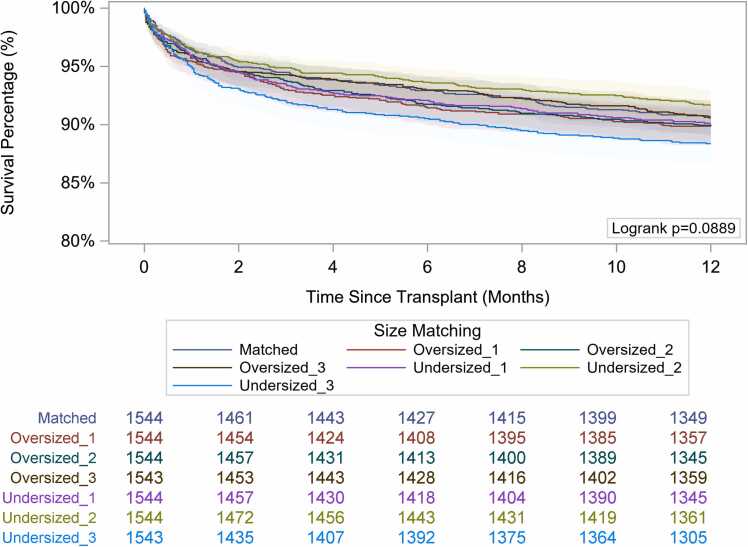
Kaplan-Meier survival curve. Kaplan-Meier curve for 1-year survival across predicted heart mass size-matching cohorts.

Unadjusted survival stratified by the degree of size mismatch is represented in Figure 1. Paired comparisons showed that compared to those who were size-matched, using severely undersized donors was associated with worse 1-year survival (88.3% [95% confidence interval [CI] 86.6-89.8] vs 90.7% [95% CI 89.1-92.0], *p* = 0.03), while oversizing was not associated with any improvement in survival (Table 4). After multivariable adjustment, only the severely undersized group had an increased risk of 1-year mortality compared to those who were size-matched (hazard ratio 1.30; CI 1.03-1.64, *p* = 0.03), while oversizing was not associated with any increased mortality risk (Table 5). This was similarly observed in the subgroup of patients bridged to transplant with HM3 (hazard ratio 2.53, 95% CI 1.37-4.69, *p* = 0.003, Table S2).

**Table 4:** One-Year Survival After Heart Transplantation

Group	1-Year survival (%)	95% Confidence interval	*p*-value (compared to matched group)
Severely undersized	88.3	86.6-89.8	0.03
Moderately undersized	91.7	90.2-92.9	0.37
Mildly undersized	90.1	88.6-91.5	0.58
Matched	90.7	89.1-92.0	n/a
Mildly oversized	89.9	88.2-91.3	0.40
Moderately oversized	90.0	88.3-91.3	0.43
Severely oversized	90.5	90.0-91.9	0.88

**Table 5:** Multivariable Cox Regression Modeling Evaluating Factors Associated With 1-Year Post-Transplant Mortality

Variable	Hazard ratio	95% Hazard ratio Confidence limits		*p*-value
Donor-recipient size-matching based on PHM ratio				
Size matched	Ref	Ref	Ref	Ref
Severely undersized	1.30	1.03	1.64	0.03
Moderately undersized	0.94	0.76	1.18	0.60
Mildly undersized	1.07	0.85	1.34	0.57
Severely oversized	0.99	0.78	1.27	0.93
Moderately oversized	1.06	0.85	1.33	0.60
Mildly oversized	1.13	0.88	1.45	0.34
*Recipient variables*				
Race				
White	Ref	Ref	Ref	Ref
Black	1.14	0.95	1.35	0.16
Hispanic	1.14	0.88	1.48	0.32
Others	1.18	0.91	1.55	0.21
Recipient age	1.03	1.02	1.03	<0.01
Heart failure etiology				
Dilated cardiomyopathy	Ref	Ref	Ref	Ref
Restrictive or hypertrophic cardiomyopathy	0.61	0.28	1.30	0.20
Ischemic cardiomyopathy	1.03	0.89	1.19	0.73
Congenital heart disease	2.26	1.27	4.05	0.01
Mean PA pressure >25 mm Hg	1.12	0.99	1.28	0.07
Pretransplant dialysis	1.88	1.37	2.60	<0.01
Diabetes	1.10	0.96	1.26	0.18
Cerebrovascular disease	1.06	0.83	1.34	0.66
Previous cardiac surgery	1.22	1.05	1.42	<0.01
Urgent listing status	1.04	0.91	1.19	0.57
Pretransplant ECMO	1.68	0.79	3.59	0.18
Pretransplant mechanical ventilation	2.03	1.09	3.80	0.03
Functional status				
Mild dysfunction	Ref	Ref	Ref	Ref
Moderate dysfunction	1.01	0.84	1.22	0.91
Severe dysfunction	1.26	1.04	1.53	0.02
Hospitalization status				
Not hospitalized	Ref	Ref	Ref	Ref
Hospitalized, non-ICU	1.01	0.84	1.21	0.93
Hospitalized, ICU	1.17	0.95	1.45	0.14
Era of transplant				
2005-2010	Ref	Ref	Ref	Ref
2011-2016	0.82	0.65	1.03	0.08
2017-2021	0.82	0.67	1.01	0.07
Pretransplant creatinine	1.14	1.05	1.24	<0.01
Pretransplant total bilirubin	1.10	1.07	1.13	<0.01
*Donor variables*				
Donor age	1.00	1.00	1.01	0.13
Sex mismatch				
Male donor to male recipient	Ref	Ref	Ref	Ref
Male donor to female recipient	1.19	0.91	1.56	0.21
Female donor to male recipient	1.03	0.86	1.24	0.76
Female donor to female recipient	1.21	1.01	1.46	0.06
Donor LVEF <50%	1.55	1.09	2.21	0.01
Ischemic time (per 1 hour increase)	1.16	1.10	1.23	<0.01

Abbreviations: ECMO, extracorporeal membrane oxygenation; ICU, intensive care unit; LVEF, left ventricular ejection fraction; PA, pulmonary artery.

**Figure 2:**
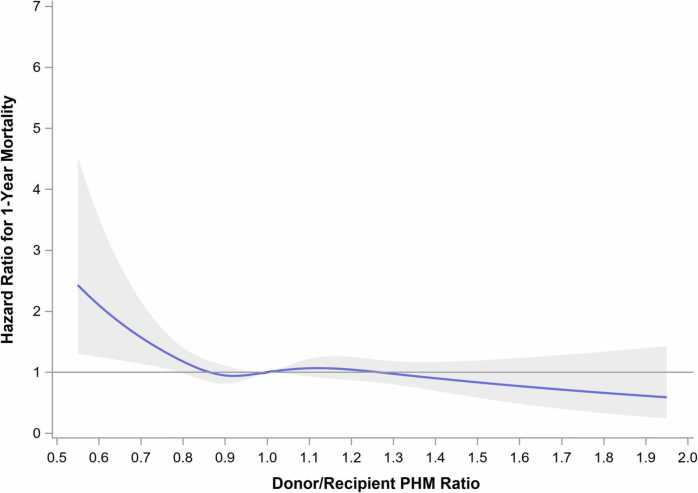
Unadjusted continuous spline. Unadjusted spine analysis for donor/recipient predicted heart mass ratio against hazard ratio for 1-year mortality (reference donor/recipient PHM ratio 1.0). PHM, predicted heart mass.

**Figure 3:**
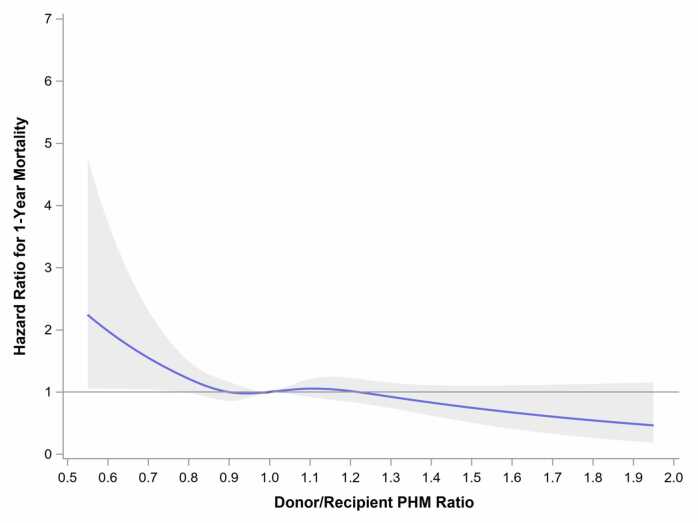
Adjusted continuous spline. Adjusted spine analysis for donor/recipient predicted heart mass ratio against hazard ratio for 1-year mortality (reference donor/recipient PHM ratio 1.0). PHM, predicted heart mass.

When the donor/recipient PHM ratio was assessed as a continuous variable, severe undersizing was again associated with an increased risk of 1-year mortality, while oversizing was not associated with any increased mortality risk, both in the unadjusted (Figure 2) and adjusted analysis (Figure 3). These findings were similarly observed in the subgroup of patients bridged to transplant with HM3 (Figures S1 and S2).

### Association between size matching and textbook outcomes

Among the 8,999 patients with complete 1-year follow-up data, the textbook outcome was achieved in 39.4% of patients, with no significant difference between groups of recipients with varying degrees of donor-recipient size mismatch (Table S3). In the multivariable analysis, neither undersizing nor oversizing was significantly associated with the odds of not achieving the composite textbook outcome, although severe undersizing approached statistical significance (odds ratio 1.17, 95% CI 0.997-1.394, *p* = 0.05, Table S4).

## Discussion

In our analysis of over 10,000 heart transplant patients who were bridged to transplantation with durable LVAD, we found that undersized donor hearts were associated with increased 1-year mortality, with an inflection point at a donor-to-recipient PHM ratio of 0.86. Importantly, for this particular population, using oversized donor hearts (PHM ratio greater than 1.01) was not associated with any survival benefit. These findings remain despite adjusting for important demographic factors, patient comorbidities, and sex matching.

PHM is a calculated metric used to determine the mass of the heart in grams and is based on prior analyses detailing the relationship between height, weight, age, sex, and the individual mass of the right and left ventricles.^5^, ^6^, ^8^ In 2019, Kransdorf et al published data from nearly 20,000 heart transplant recipients demonstrating the superiority of the donor-to-recipient PHM ratio over other metrics of size matching for predicting mortality following HTX.^1^ In this seminal article, the authors found an increased risk of mortality in patients with a donor-recipient PHM ratio of less than 0.86. Importantly, they also found that 32% of heart offers rejected due to donor size had PHM ratios greater than 0.86, while just 14% of accepted donor hearts were below the 0.86 threshold. Given the promising mortality benefits and opportunity for expansion of the acceptable donor pool, these data have been subsequently validated in a number of large-registry studies, and the International Society for Heart and Lung Transplantation has officially recommended the use of PHM calculations in clinical decision-making.

While size matching is intuitively important for post-transplant hemodynamic stability, the PHM calculation does not take into account many important factors that can affect pre- and post-transplant hemodynamic stability in individual patients. Since heart transplant allocation changes in 2018, the profile of LVAD patients being bridged to heart transplant has changed dramatically, with an increase in the use of marginal donors (high-risk donors, increased ischemic times), and subsequent increases in mortality.^9^ HTX following LVAD is associated with greater perioperative risk due to the nature of redo sternotomy cardiac surgery, including longer cardiopulmonary bypass duration, more blood transfusions, prolonged intubation, and longer intensive care unit stay.^10^, ^11^ For these reasons, there may be a theoretical benefit to oversizing donor hearts in these recipients to offset the expected postoperative vasoplegia with improved cardiac output; however, our data show that oversizing donor hearts in post-LVAD HTX does not improve 1-year mortality.^12^

While numerous studies have reported the superiority of PHM for size matching in HTX, a recently published manuscript by Siddegowda-Bangalore et al sought to determine if PHM was the optimal size-matching metric for patients bridged with durable LVADs. In doing so, the authors also found that oversized donor hearts do not result in improved 1-year outcomes.^13^ By investigating a more contemporary patient population with a significant proportion of patients bridged with HM3, which is currently the only Food and Drug Administration-approved durable LVAD available, our results bear clinical relevance to the current practice of HTX. We also treated the donor-recipient PHM ratio as a continuous, nonlinear variable in addition to arbitrarily defined groups. This allowed us to more accurately assess the effect of varying degrees of donor-recipient size mismatch. Furthermore, to capture the demonstrated perioperative risk following LVAD explant, we included an analysis of “textbook outcomes.” In doing so, we demonstrated that neither undersizing nor oversizing has a significant effect on the composite outcome, including hospital length of stay, ejection fraction, functional status, and perioperative and 1-year morbidity, although there was a trend toward worse outcomes in the severely undersized cohort. This may be related to the fact that approximately 15% of the initial cohort was excluded from this analysis due to a lack of 1-year follow-up data, resulting in reduced statistical power to detect a significant difference associated with severe undersizing. Nevertheless, the lack of significant differences in our composite outcome provides further support in favor of liberalization of size-matching criteria for expanded donor usage, beyond a singular focus on 1-year mortality.

For patients on the heart transplant waitlist, there is a growing body of evidence to suggest expanded use of “marginal” or higher-risk donor organs. Han et al recently explored the impact of higher-risk donor usage, including undersized donor hearts, in recipients bridged to transplant with MCS.^14^ Their results show that in patients bridged with durable LVADs, survival is best with receipt of a standard-risk donor heart. This finding is in contrast to patients on other forms of temporary MCS, for whom high-risk donors have no impact on short-term outcomes and actually improve survival when compared to remaining on the waitlist. Our study provides further evidence that LVAD patients in particular benefit from the optimization of donor-recipient matching. PHM provides an improvement in this optimization over prior techniques, such as height/weight matching, but is still a calculated value from readily available demographic information. With this simplicity and ease comes a lack of granularity, and some have argued that even better metrics and individual assessments are needed, such as the utilization of donor CT imaging.^15^, ^16^, ^17^ However, PHM is a universally available, easily calculated metric, and provides a balance between precision size-matching and optimal utilization of grafts. Our data add to the existing evidence that undersized donor hearts perform poorly, while providing further evidence that, even in recipients with a theoretical benefit for oversized donor hearts, size-matched donor hearts (PHM 0.86-1.01) perform just as well.^12^, ^14^, ^18^

### Limitations

Despite the use of data from a robust national transplant registry, our study evaluates outcomes among patients who have received a donor heart and therefore inherently excludes unused donor hearts as well as waitlist candidates who do not survive to transplantation. Data regarding post-transplant MCS and primary graft dysfunction were not available in the UNOS registry, which are important factors in assessing recipient outcomes, and reflect the importance of including analysis of textbook outcomes in this study. In the analysis of textbook outcomes, complete data to ascertain this composite end-point were only available in approximately 85% of patients, as 1,807 patients did not have 1-year follow-up data and were thus excluded from analysis. Additionally, this composite textbook outcome was defined based on previous publications, and no universally accepted definition currently exists. We divided patients into 7 equal groups based on donor-to-recipient PHM ratio. The subsequent cut points used for each group are thus arbitrary. Further studies are needed to define the optimal threshold for size matching better. Last, we excluded patients with previous heart transplants, multiorgan transplants, and a body mass index >40. Our study findings therefore must be extrapolated with caution to these patient populations.

## Conclusions

This analysis of over 10,000 adult heart transplant recipients who were bridged to transplantation with durable LVADs demonstrated no benefit to oversizing donor hearts, as compared to size-matched references, using the PHM donor-recipient ratio. In line with prior analyses of heart transplant recipients, recipients bridged with LVADs had a similar increase in 1-year mortality when undersized donors were used. The results of this study add to the existing literature regarding heart transplant size-matching and may be considered for a more liberal use of slightly undersized donor hearts (PHM 0.87-0.96) in recipients with LVADs.

## Author contributions

Ross Michael Reul: Conceptualization, Writing – Original Draft, Writing – Review and Editing. Qiudong Chen: Conceptualization, Methodology, Formal Analysis, Writing – Review and Editing, Resources. Joshua Chan: Conceptualization, Methodology, Writing – Review and Editing, Supervision, Project Administration.

## Disclosure statement

The authors declare that they have no known competing financial interests or personal relationships that could have appeared to influence the work reported in this paper.

## Appendix A Supplementary material

.

Supplemental Figure 1. Unadjusted continuous spline for HM3 cohort. Unadjusted spine analysis for donor/recipient predicted heart mass ratio against hazard ratio for 1-year mortality (reference donor/recipient PHM ratio 1.0) in recipients bridged to transplantation with HeartMate 3 LVAD/recipient predicted heart mass ratio against hazard ratio for 1-year mortality (reference donor/recipient PHM ratio 1.0) in recipients bridged to transplantation with HeartMate 3 LVAD. HM3, HeartMate 3; LVAD, left ventricular assist device; PHM, predicted heart mass.

## Footnotes

Appendix A Supplementary data associated with this article can be found in the online version at doi:10.1016/j.jhlto.2024.100150.
