# Supplementary material for: Application of donor predicted heart mass in heart transplant recipients with left ventricular assist device
Source: JHLT Open. 2024 Aug 22;6:100150. doi: 10.1016/j.jhlto.2024.100150 (PMC11935510; doi:10.1016/j.jhlto.2024.100150)
Supplement: Supplementary file 1 — Supplementary material. [file mmc1.docx]

**Supplemental Table 1: Cutoff values for donor to recipient predicated heart mass ratio for the heartmate 3 only cohort**

| **Group** | **Number of Patients** | **Median Donor to Recipient PHM Ratio** | **Mean Donor to Recipient PHM Ratio** | **Minimum Donor to Recipient PHM Ratio** | **Maximum Donor to Recipient PHM Ratio** |
| --- | --- | --- | --- | --- | --- |
| Severely undersized | **181** | 0.8077234 | 0.7984699 | 0.6360402 | 0.8535502 |
| Moderately undersized | **181** | 0.8800790 | 0.8802586 | 0.8544110 | 0.9039132 |
| Mildly undersized | **181** | 0.9235369 | 0.9233592 | 0.9040734 | 0.9460373 |
| Matched | **181** | 0.9686044 | 0.9689421 | 0.9461650 | 0.9932135 |
| Mildly oversized | **181** | 1.0167587 | 1.0179266 | 0.9942678 | 1.0438760 |
| Moderately oversized | **181** | 1.0851541 | 1.0890381 | 1.0439924 | 1.1361567 |
| Severely oversized | **181** | 1.2137800 | 1.2528272 | 1.1378997 | 1.67712 |

**Supplemental Table 2. Multivariable Cox regression modeling evaluating factors associated with 1-year post-transplant mortality in the subgroup of patients bridged to transplant with a HeartMate 3 device**

| **Variable** | **Hazard Ratio** | **95% Hazard Ratio Confidence Limits** | | **P value** |
| --- | --- | --- | --- | --- |
| Donor recipient size matching based on PHM ratio |  |  | |  |
| Size matched | Ref | Ref | Ref | Ref |
| Severely undersized | 2.534 | 1.371 | 4.686 | 0.0030 |
| Moderately undersized | 1.603 | 0.830 | 3.097 | 0.1601 |
| Mildly undersized | 1.379 | 0.665 | 2.858 | 0.3880 |
| Severely oversized | 1.552 | 0.810 | 2.971 | 0.1850 |
| Moderately oversized | 1.362 | 0.701 | 2.643 | 0.3617 |
| Mildly oversized | 1.730 | 0.856 | 3.496 | 0.1266 |
| Recipient variables | | | | |
| Race |  |  |  |  |
| White | Ref | Ref | Ref | Ref |
| Black | 1.188 | 0.743 | 1.900 | 0.4706 |
| Hispanic | 1.188 | 0.743 | 1.900 | 0.4706 |
| Others | 1.554 | 0.692 | 3.491 | 0.2857 |
| Recipient age | 1.039 | 1.020 | 1.058 | <.0001 |
| Heart failure etiology |  |  |  |  |
| Dilated cardiomyopathy | Ref | Ref | Ref | Ref |
| Restrictive or hypertrophic cardiomyopathy | 1.385 | 0.144 | 13.307 | 0.7776 |
| Ischemic cardiomyopathy | 1.227 | 0.805 | 1.870 | 0.3407 |
| Congenital heart disease | 2.776 | 0.673 | 11.462 | 0.1581 |
| Mean PA pressure > 25mmHg | 1.315 | 0.885 | 1.956 | 0.1755 |
| Pretransplant dialysis | 2.430 | 0.802 | 7.358 | 0.1164 |
| Diabetes | 1.252 | 0.917 | 1.709 | 0.1566 |
| Cerebrovascular disease | 1.398 | 0.656 | 2.979 | 0.3856 |
| Previous cardiac surgery | 1.377 | 0.892 | 2.126 | 0.1489 |
| Urgent listing status | 1.039 | 0.756 | 1.429 | 0.8126 |
| Pre-transplant ECMO | 1.970 | 0.170 | 22.876 | 0.5879 |
| Pre-transplant mechanical ventilation | 0.000 | 0.000 | 0.000 | <.0001 |
| Functional status |  |  |  |  |
| Mild dysfunction | Ref | Ref | Ref | Ref |
| Moderate dysfunction | 1.011 | 0.609 | 1.677 | 0.9677 |
| Severe dysfunction | 1.221 | 0.710 | 2.101 | 0.4698 |
| Hospitalization status |  |  |  |  |
| Not hospitalized | Ref | Ref | Ref | Ref |
| Hospitalized, non-ICU | 0.739 | 0.391 | 1.397 | 0.3526 |
| Hospitalized, ICU | 0.897 | 0.456 | 1.766 | 0.7536 |
| Pre-transplant creatinine | 1.394 | 1.071 | 1.815 | 0.0136 |
| Pre-transplant total bilirubin | 1.234 | 1.124 | 1.355 | <.0001 |
| Donor Variables | | | | |
| Donor age | 1.007 | 0.992 | 1.023 | 0.3670 |
| Sex mismatch |  |  |  |  |
| Male donor to male recipient | Ref | Ref | Ref | Ref |
| Male donor to female recipient | 1.142 | 0.548 | 2.378 | 0.7225 |
| Female donor to male recipient | 0.721 | 0.405 | 1.286 | 0.2677 |
| Female donor to female recipient | 1.806 | 1.098 | 2.969 | 0.0198 |
| Donor LVEF<50% | 1.697 | 0.674 | 4.271 | 0.2614 |
| Ischemic time (per 1 hour increase) | 1.044 | 0.890 | 1.225 | 0.5944 |

**Supplemental Table 3: Percentage of recipients who achieved textbook outcome, stratified by the degree of donor recipient size mismatch based on predicted heart mass.**

|  | **Severely undersized**  **(n=1263)** | **Moderately undersized**  **(n=1292)** | **Mildly undersized (n=1276)** | **Matched (n=1289)** | **Mildly oversized (n=1290)** | **Moderately oversized (n=1306)** | **Severely oversized**  **(n=1283)** | **P value** |
| --- | --- | --- | --- | --- | --- | --- | --- | --- |
| Textbook outcome achieved | 37.1 (469) | 38.7 (500) | 40.4 (515) | 39.9 (514) | 42.2 (544) | 39.0 (509) | 38.6 (495) | 0.22 |

Analysis was performed in a subgroup of 8999 patients with complete 1-year follow up.

Textbook outcome was defined as as post-transplant hospital length of stay < 30 days; ejection fraction greater than 50% during 1-year follow-up; functional status 80% to 100% at 1 year; freedom from acute rejection, dialysis, and stroke during the index hospitalization; and freedom from graft failure, dialysis, rejection, retransplantation, or mortality during the first year after transplantation

**Supplemental Table 4. Results from the multivariable logistic regression to determine the association between size matching and the likelihood of not achieving textbook outcome**

| **Group** | **Odds Ratio** | **95%** | | **P value** |
| --- | --- | --- | --- | --- |
|  |  | **Confidence Limits** | |  |
| **Size matched** | Ref | Ref | Ref | Ref |
| **Severely oversized** | 1.0661 | 0.9106 | 1.2482 | 0.4259 |
| **Moderately oversized** | 1.0193 | 0.8772 | 1.1845 | 0.8028 |
| **Mildly oversized** | 0.9194 | 0.7966 | 1.0611 | 0.2505 |
| **Mildly undersized** | 1.0088 | 0.8826 | 1.1530 | 0.8982 |
| **Moderately undersized** | 1.0533 | 0.9105 | 1.2186 | 0.4847 |
| **Severely undersized** | 1.1788 | 0.9970 | 1.3938 | 0.0543 |

Analysis was performed in a subgroup of 8999 patients with complete 1-year follow up.

Textbook outcome was defined as as post-transplant hospital length of stay < 30 days; ejection fraction greater than 50% during 1-year follow-up; functional status 80% to 100% at 1 year; freedom from acute rejection, dialysis, and stroke during the index hospitalization; and freedom from graft failure, dialysis, rejection, retransplantation, or mortality during the first year after transplantation
