# Supplementary figures and images for: Application of donor predicted heart mass in heart transplant recipients with left ventricular assist device
Source: JHLT Open. 2024 Aug 22;6:100150. doi: 10.1016/j.jhlto.2024.100150 (PMC11935510; doi:10.1016/j.jhlto.2024.100150)

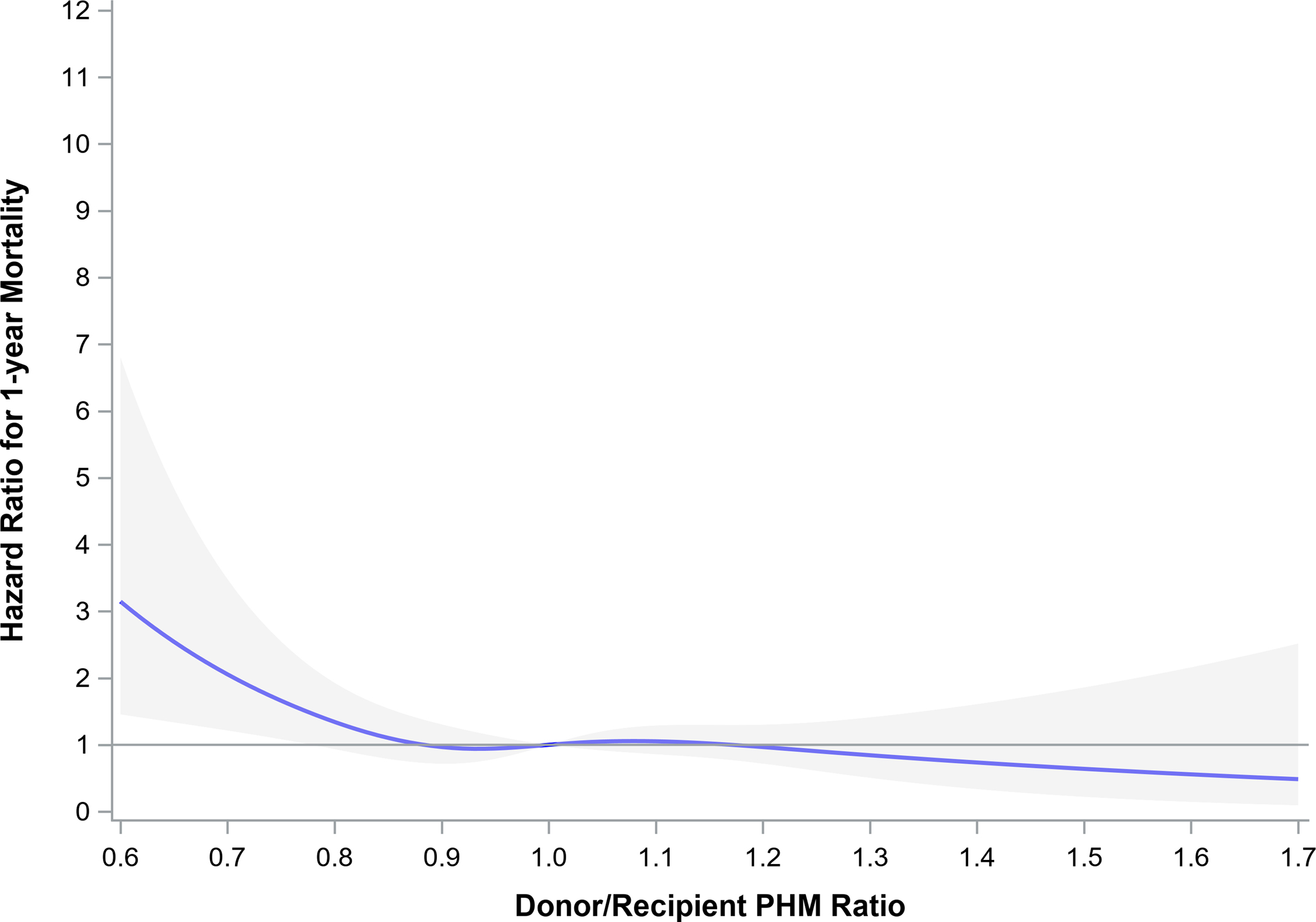

Supplement: Supplementary file 2 — Supplemental Figure 1. Unadjusted continuous spline for HM3 cohort. Unadjusted spine analysis for donor/recipient predicted heart mass ratio against hazard ratio for 1-year mortality (reference donor/recipient PHM ratio 1.0) in recipients bridged to transplantation with HeartMate 3 LVAD/recipient predicted heart mass ratio against hazard ratio for 1-year mortality (reference donor/recipient PHM ratio 1.0) in recipients bridged to transplantation with HeartMate 3 LVAD. HM3, HeartMate 3; LVAD, left ventricular assist device; PHM, predicted heart mass. [file mmc2.jpg]

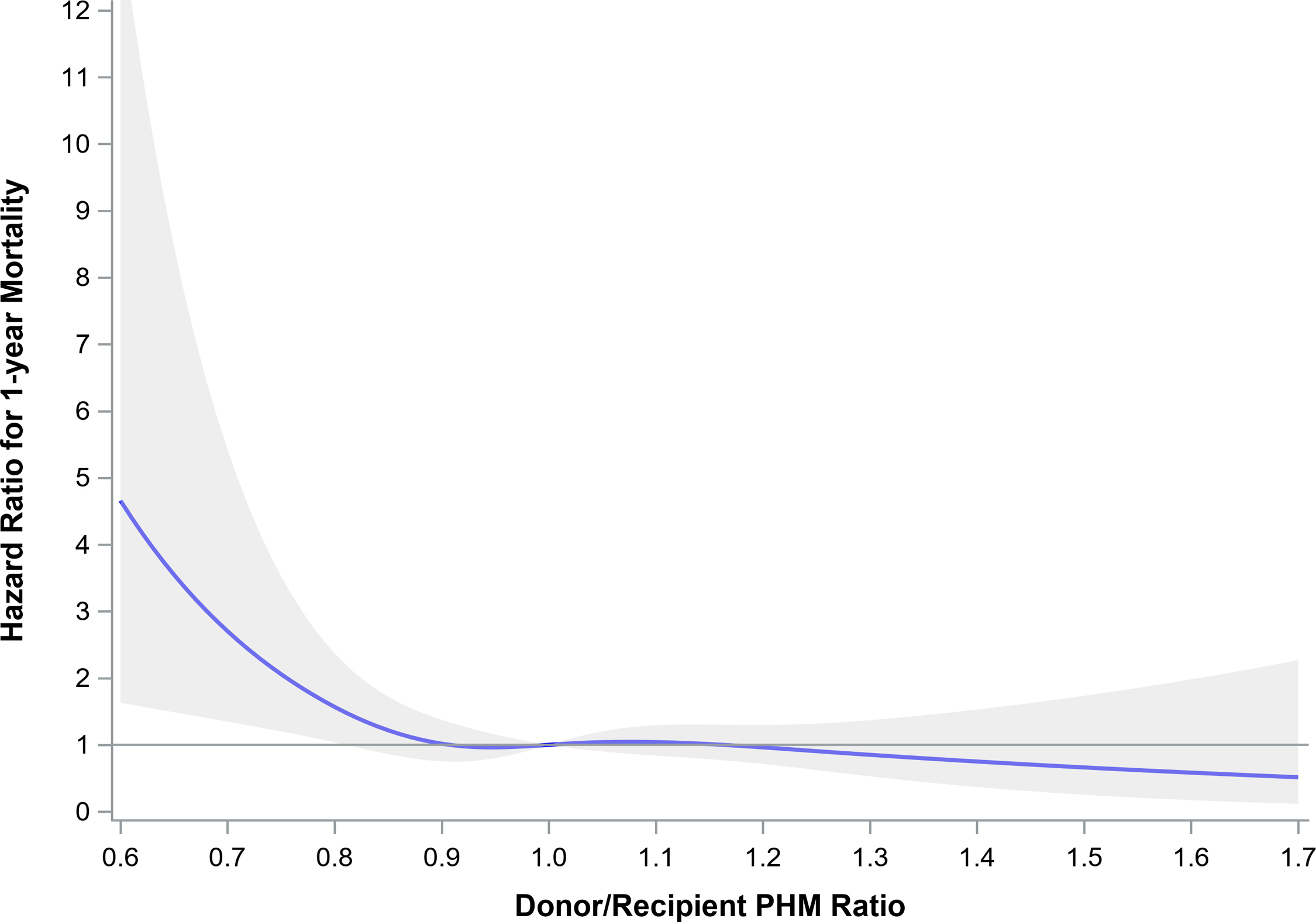

Supplement: Supplementary file 3 — Supplemental Figure 1. Unadjusted continuous spline for HM3 cohort. Unadjusted spine analysis for donor/recipient predicted heart mass ratio against hazard ratio for 1-year mortality (reference donor/recipient PHM ratio 1.0) in recipients bridged to transplantation with HeartMate 3 LVAD/recipient predicted heart mass ratio against hazard ratio for 1-year mortality (reference donor/recipient PHM ratio 1.0) in recipients bridged to transplantation with HeartMate 3 LVAD. HM3, HeartMate 3; LVAD, left ventricular assist device; PHM, predicted heart mass. [file mmc3.jpg]
